# All-Optically Tunable Broadband Terahertz Modulators Based on Large-Area WSe_2_/Si Heterojunction Grown by Sputtering and Selenization

**DOI:** 10.3390/mi17080952

**Published:** 2026-08-11

**Authors:** Shijie Lu, Deqiang Han, Junjie Song, Yizhen Lin, Han Xu, Guangjun Lu

**Affiliations:** 1 College of Science, Harbin University of Science and Technology, Harbin 150080, China; 2 Department of Electronic Science and Technology, School of Electrical and Electronic Engineering, Harbin University of Science and Technology, Harbin 150080, China; 3 Guangxi Key Laboratory of Brain-Inspired Computing and Intelligent Chips, School of Electronic and Information Engineering, Guangxi Normal University, Guilin 541004, China

**Keywords:** 2D transition metal selenides, heterojunction, optical excitation, THz modulation, modulation depth

## Abstract

Effective terahertz (THz) modulation remains a critical challenge for advancing THz technology. Recently, two-dimensional (2D) materials integrated with high-resistance silicon (Si) have emerged as promising heterojunction platforms for THz control. In this paper, we experimentally demonstrate an all-optically controlled THz modulator based on a large-area WSe_2_/Si heterojunction, fabricated by magnetron sputtering of tungsten on Si followed by vacuum selenization. The device exhibits broadband modulation across 0.1–1.1 THz, achieving a modulation depth of 72% at a pump power of 1500 mW/cm^2^, significantly higher than that of bare Si or pristine WSe_2_ films. Moreover, we systematically analyze the underlying mechanism responsible for this enhanced performance. This study presents an effective and scalable strategy for THz modulation using 2D material/Si heterojunctions.

## 1. Introduction

Terahertz (THz) waves have garnered considerable interest due to their unique advantages in medical imaging [1,2], communications [3], sensing [4], molecular spectroscopy [5,6,7], and other promising applications. Although significant progress has been made in THz generators and detectors, the development of functional devices, especially modulators, remains a bottleneck [6]. THz modulators, which enable dynamic control over amplitude, phase, and polarization [8], have been realized using diverse materials [7,9,10,11,12,13,14,15,16,17]. However, most existing designs suffer from high insertion losses, stringent operating requirements, and limited modulation range, hindering practical deployments and applications. As THz technology matures, there is a growing demand for modulators with higher modulation depth and broader bandwidth [18].

Current THz modulation strategies rely primarily on optical, electrical, or thermal stimuli [19]. Among these, all-optical modulation excels in both modulation depth and speed, positioning heterojunction-based all-optical THz modulators as a promising avenue [20]. In this context, two-dimensional (2D) materials have emerged as attractive candidates owing to their excellent optoelectronic properties. Compared with conventional semiconductors, 2D materials offer higher carrier mobility and shorter relaxation times, which are critical parameters for device performance [21]. For example, Weis et al. first demonstrated high-efficiency THz modulation in graphene under femtosecond laser pumping [22]. Subsequently, Wen et al. proposed optically active graphene-on-germanium modulators [23]. Nevertheless, graphene’s intrinsic zero bandgap restricts its further development in the THz applications. Transition metal dichalcogenides (TMDCs), with bandgaps of 1–3 eV, overcome this limitation and hold great promise for THz devices [24,25]. Moreover, TMDCs can form type-II heterojunctions with traditional semiconductors, alleviating the weak light absorption inherent to their atomic-scale thickness [26,27]. For instance, Chen et al. first reported a MoS_2_/Si heterostructure THz modulator grown via chemical vapor deposition (CVD) [8]. Subsequently, Cao et al. improved the modulation depth of similar devices through annealing [28]. To further enhance modulation depth, Zheng et al. introduced an optically pumped MoS_2_-Si heterostructure metasurface THz modulator with monolayer MoS_2_ grown by high-pressure CVD [29]. Additionally, Qiao et al. demonstrated a high-performance MoTe_2_/Si all-optically controlled THz modulator fabricated by spin-coating [30]. Alka Jakhar et al. fabricated a PtSe_2_/Si-based THz modulator using a combination strategy of both CVD and physical vapor deposition (PVD) methods [31]. Lv et al. developed a broadband THz modulator based on an optically controlled GeSe_2_/Si heterojunction via magnetron sputtering and vacuum selenization [32]. Despite these advances, current THz modulators still face key challenges, including the fabrication of large-area continuous films and low modulation depth, which severely hinder practical applications [33,34].

Among TMDCs, WSe_2_ is particularly attractive for THz modulation due to its direct bandgap, enabling efficient photoexcitation and strong light–matter interaction [25]. More importantly, WSe_2_ forms a type-II heterojunction with Si, featuring conduction- and valence-band offsets of ~0.05 eV and ~0.13 eV, respectively [35]. This alignment promotes efficient spatial separation of photogenerated electron-hole pairs at the interface, suppressing carrier recombination and prolonging carrier lifetime, and thus enhancing photoconductivity and modulation depth. These interface-induced benefits are directly evidenced by time-resolved THz emission spectroscopy, which reveals that the built-in electric field accelerates carrier migration and amplifies photocurrent by nearly an order of magnitude [36]. Compared with MoS_2_ (indirect bandgap ~1.29 eV) and MoTe_2_ (narrower bandgap ~1.0 eV), WSe_2_ offers superior band alignment with Si and stronger valley-spin coupling, favorable for photocarrier manipulation. Previous experiments have confirmed that monolayer WSe_2_ on Si increases THz modulation depth by approximately 40% relative to bare Si [37]. From a fabrication standpoint, magnetron sputtering followed by vacuum selenization yields large-area, continuous, highly crystalline WSe_2_ films with excellent uniformity, compatible with standard semiconductor processing for scalable production [38]. These combined merits motivate our development of a WSe_2_/Si heterojunction-based THz modulator.

In this paper, we demonstrate an optically controlled THz modulator based on a large-area WSe_2_/Si heterojunction fabricated by magnetron sputtering followed by vacuum selenization. Under 850 nm continuous-wave (CW) laser pumping at 1500 mW/cm^2^, the device exhibits a broadband response from 0.1 to 1.1 THz and achieves a maximum modulation depth of 72% at 0.5 THz, significantly outperforming bare Si, single WSe_2_ structures, and graphene-based modulators. Therefore, this work provides a feasible pathway for large-area 2D TMDC-based THz modulation.

## 2. Preparation and Characterization

### 2.1. Fabrication of WSe_2_/Si Heterojunction

**Figure 1:**
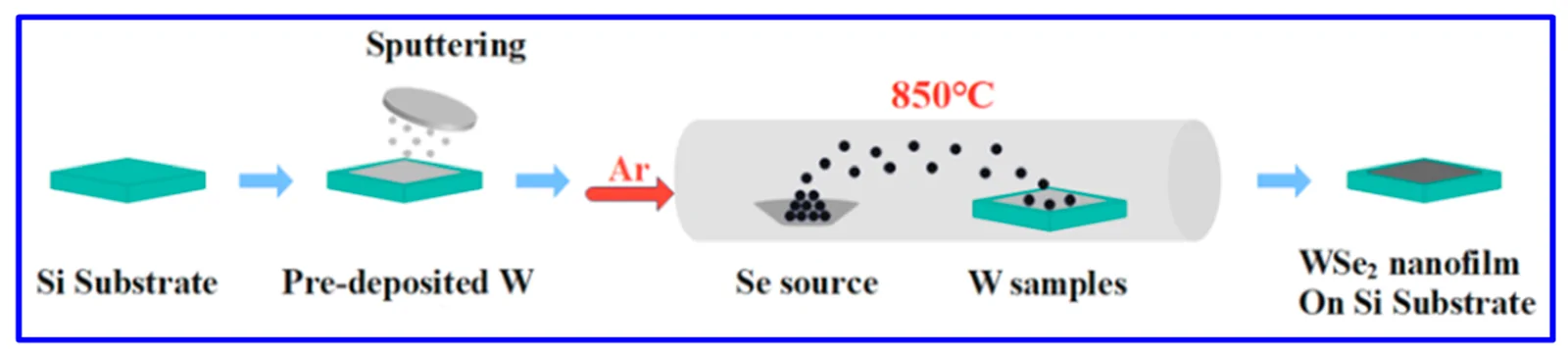
Preparation flow of the WSe_2_/Si heterojunction.

WSe_2_/Si heterojunction was fabricated via a two-step process, as illustrated in Figure 1. First, tungsten (W) films were deposited onto a single-side-polished 500 μm-thick high-resistance Si substrate (110) by magnetron sputtering. Prior to deposition, the substrates were cleaned with ethanol and deionized water. The sputtering parameters for the tungsten film are summarized in Table 1. Subsequently, the as-deposited W film and high-purity selenium (Se) powder (99.99%) were sealed in an evacuated quartz tube and heated to 850 °C at a ramp rate of 5 °C min^−1^ for 3 h to promote selenization according to W + 2Se → WSe_2_. To prevent oxidation, Ar gas (30 sccm) was introduced into the tube throughout the reaction. After selenization, the sample was naturally cooled to room temperature inside the tube furnace before removal. Detailed reaction parameters are listed in Table 2.

### 2.2. Characterization of WSe_2_/Si Heterojunction

**Figure 2:**
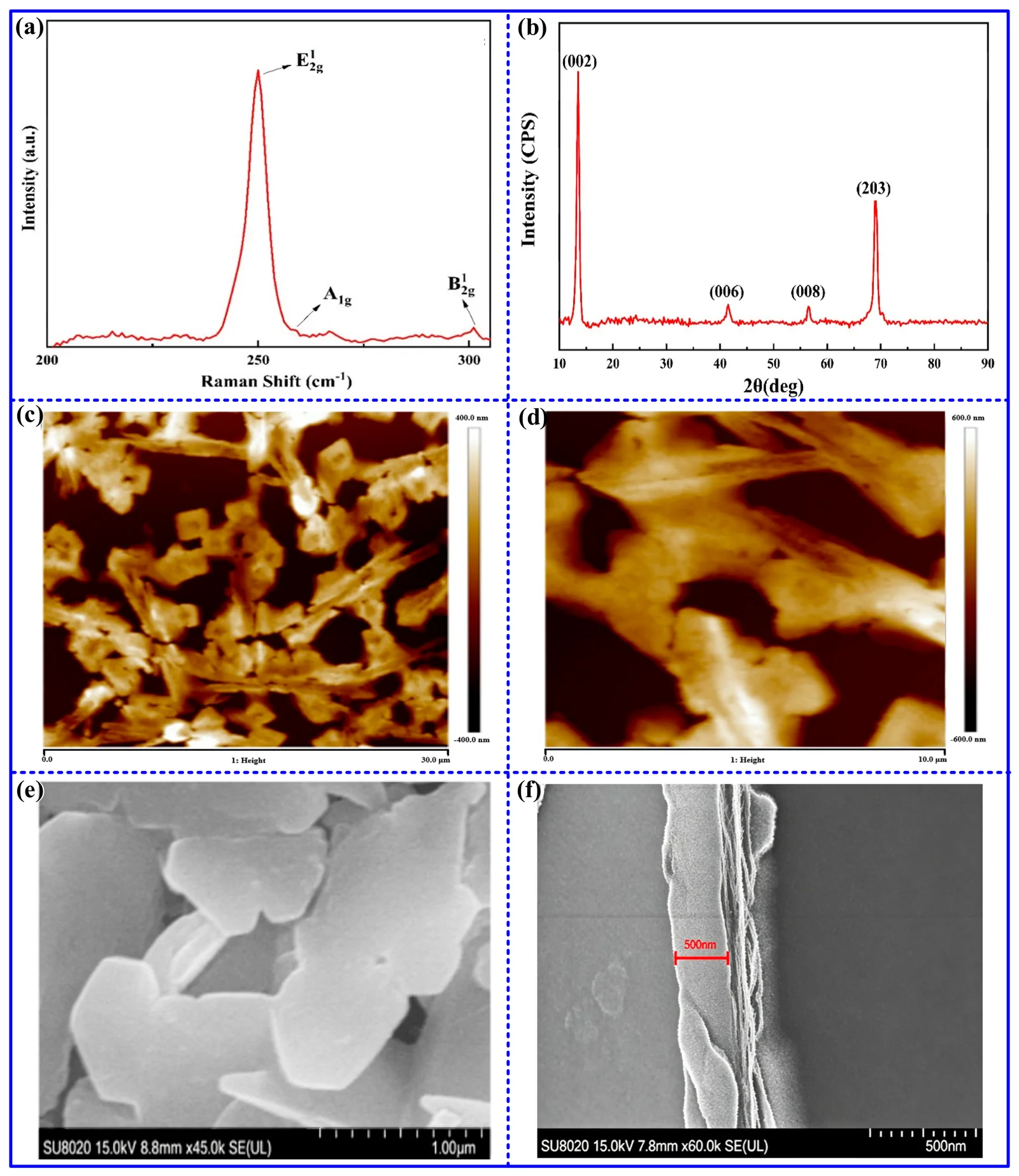
Characterization of the fabricated WSe_2_/Si samples: (**a**) Raman spectrum, (**b**) XRD pattern, (**c**) AFM image (30 μm scale), (**d**) AFM image (10 μm scale), (**e**) SEM surface image, and (**f**) cross-sectional SEM image.

The as-prepared WSe_2_/Si heterojunction was systematically characterized by Raman spectroscopy, X-ray diffraction (XRD), atomic force microscopy (AFM), and scanning electron microscopy (SEM), as shown in Figure 2. Raman spectrum (Figure 2a) reveals a prominent peak at approximately 250 cm^−1^, assigned to the $E_{2g}^{1}$ mode characteristic of few-layer WSe_2_. The XRD pattern (Figure 2b) exhibits sharp diffraction peaks indexed to the (002), (006), (008), and (203) planes, confirming a highly c-axis-oriented WSe_2_ film with good crystallinity and no detectable secondary orientations. AFM topography was acquired in tapping mode over scan areas of 30 μm × 30 μm and 10 μm × 10 μm (Figure 2c,d). Raw data were processed only with standard flattening routines to remove background tilt. The AFM images reveal a pinhole-rich surface with unevenly distributed WSe_2_. SEM micrographs, obtained at 15 kV, show densely packed WSe_2_ grains larger than 1 μm with multidirectional growth in plan-view (Figure 2e), while the cross-sectional view indicates a uniform film thickness of approximately 500 nm (Figure 2f). Collectively, these results confirm the successful growth of a dense, highly crystalline, and well-adhered WSe_2_ film on Si via magnetron sputtering followed by selenization, providing a solid foundation for the THz modulation studies presented below.

## 3. Results and Discussion

**Figure 3:**
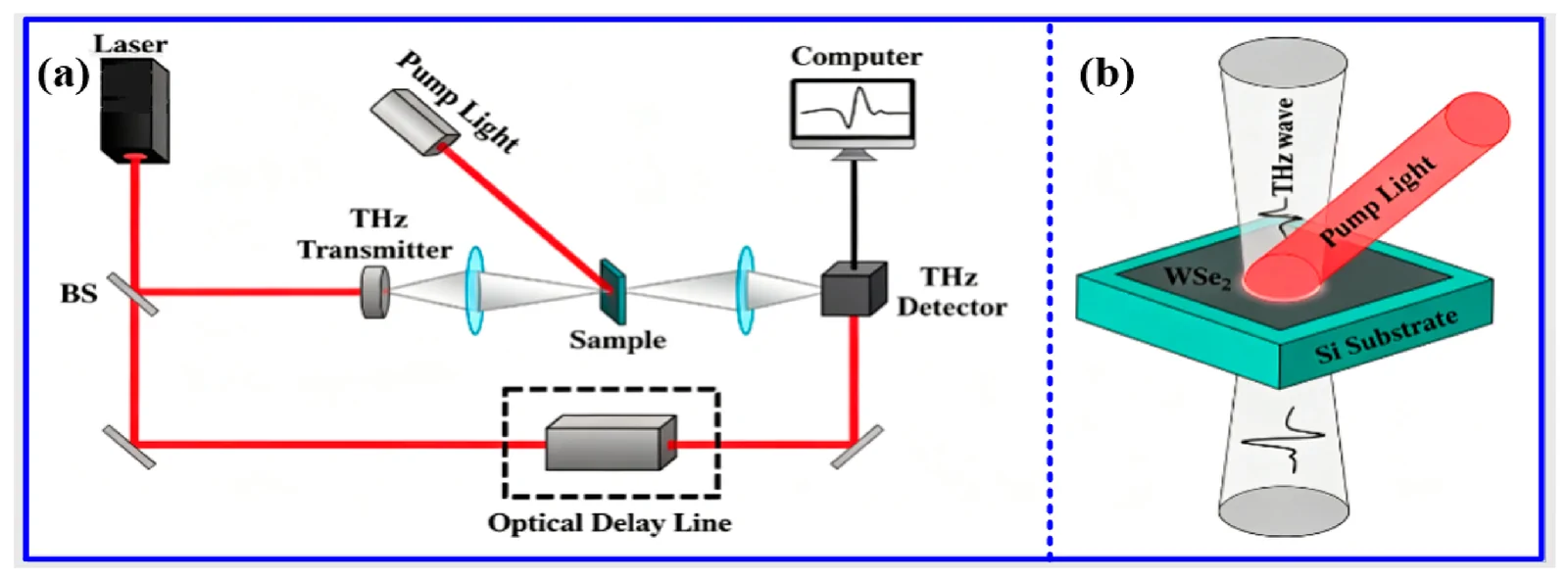
THz characterization of the prepared modulator: (**a**) schematic diagram of the THz-TDS system integrated with an 850 nm CW laser source and (**b**) schematic of the WSe_2_/Si heterojunction THz modulator.

The THz modulation performance of the WSe_2_/Si heterojunction was characterized using a home-built THz time-domain spectroscopy (THz-TDS) system based on asynchronous optical sampling, as schematically shown in Figure 3a. The system employs a MaiTai SP femtosecond laser (90 fs pulse duration, 800 nm center wavelength, and 1 kHz repetition rate) as the source, with photoconductive antennas for THz generation and detection, a delay stage, and a lock-in amplifier. The emitted THz beam is collected and collimated by four off-axis parabolic mirrors. An 850 nm CW laser serves as the pump source to enable efficient interband absorption, as its photon energy exceeds the bandgaps of both WSe_2_ and Si [39]. The pump spot is carefully aligned to fully overlap with the THz beam on the modulator surface (Figure 3b). Since the pump is a CW source, it does not generate impulsive THz emission via optical rectification or transient photocurrent. Instead, it merely establishes a quasi-static photogenerated carrier distribution that modifies the complex conductivity and attenuates the transmitted THz probe pulse. Any incoherent steady-state background from the CW pump lies outside the THz range or is suppressed by the lock-in amplifier synchronized to the probe beam modulation. Furthermore, the absence of synchronous pump-probe delay gating ensures that any residual emits. Thus, the observed modulation purely reflects the pump-induced conductivity change, free from spurious THz emission or background artifacts.

**Figure 4:**
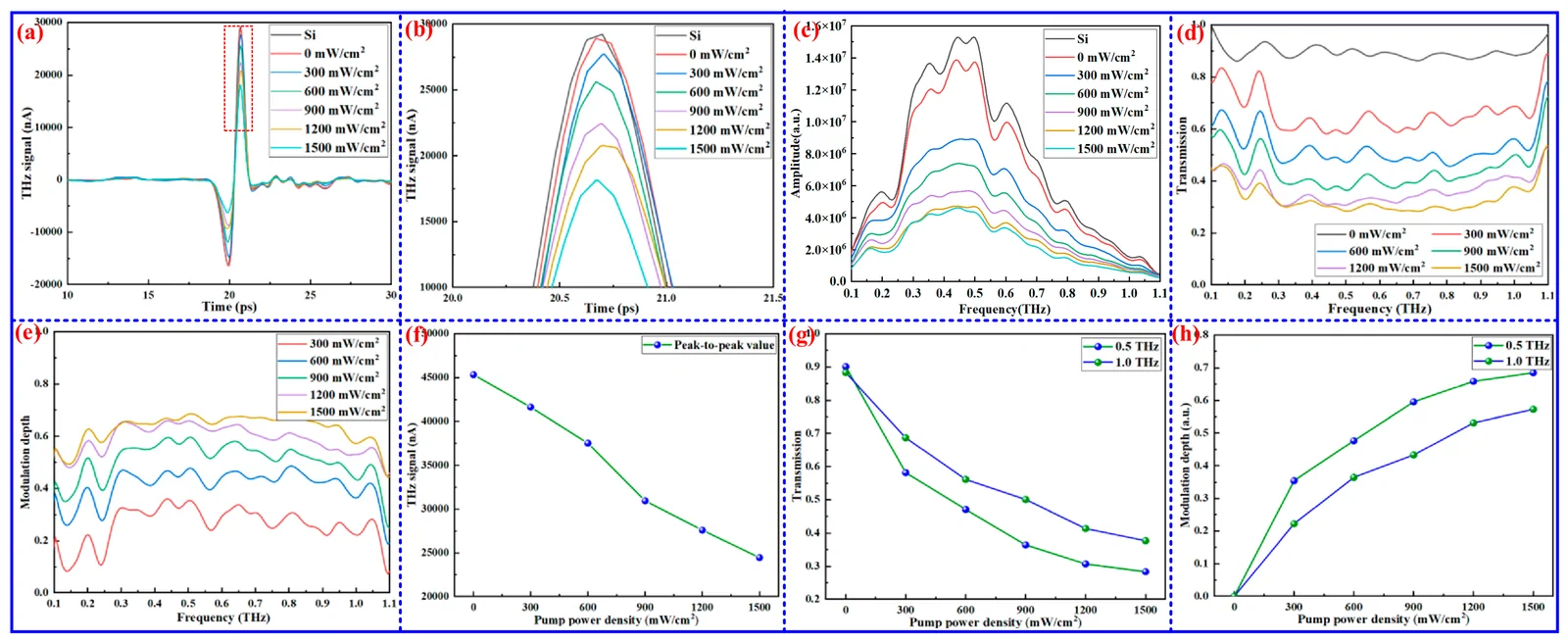
Transmission performance of the WSe_2_/Si heterojunction THz modulator: (**a**) time-domain spectra, (**b**) frequency-domain spectra, (**c**) modulation depth, (**d**) peak-to-peak values versus pump power, (**e**) transmission intensities at 0.5 and 1.0 THz versus different pump powers, and (**f**) modulation depths at 0.5 and 1.0 THz versus different pump powers. (**g**) Normalized transmission intensity versus pump power at 0.5 and 1.0 THz. (**h**) Modulation depth (MD) versus pump power at the same frequencies.

Figure 4a presents the time-domain THz transmission signals of the WSe_2_/Si heterojunction modulator with and without pump irradiation, using the bare Si substrate as a reference (not free space). Although the 500-μm Si wafer introduces a bulk delay of several picoseconds relative to air, this delay is identical for the reference and sample and cancels out in direct comparison. Without pumping, a slight amplitude attenuation and a negligible time shift (<0.05 ps, within the system jitter and experimental resolution) are observed between the heterojunction and the bare Si reference (Figure 4b, confirming that the 500 nm WSe_2_ overlayer introduces no appreciable phase lag. Therefore, this combination of small attenuation and the negligible delay unequivocally substantiates the successful formation of the WSe_2_ thin film on Si. As the pump power increases from 0 to 1500 mW/cm^2^, the THz amplitude decreases monotonically, indicating a clear pump-power-dependent conductivity modulation. The corresponding frequency-domain spectra, obtained by fast Fourier transform of the time-domain waveforms, show consistent trends (Figure 4c). To avoid numerical artifacts, all time-domain signals were zero-padded and multiplied by a symmetric window function with identical parameters, ensuring reliable relative comparisons. To further quantify the frequency-dependent behavior, normalized transmission spectra were extracted using:

(1) $$T(\omega )=E_{\mathrm{sam}}(\omega )/E_{\mathrm{ref}}(\omega )=A(\omega )e^{i\varphi (\omega )}$$

where $E_{\mathrm{ref}}\left(\omega \right)$ and $E_{\mathrm{sam}}(\omega )$ are the Fourier transforms of the reference and sample time-domain signals, and A(ω) and φ(ω) are the amplitude and phase of transmission coefficients. Figure 4d displays the normalized transmission spectra from 0.1 to 1.1 THz under various pump powers. Without excitation, THz waves readily penetrate the modulator. As the pump power increases, the normalized transmission intensity decreases markedly across the entire frequency range, indicating a broadband modulation. For instance, the average normalized transmission drops from 0.92 to 0.28 over 0.1–1.1 THz as the pump power increases from 0 to 1200 mW/cm^2^. At 1500 mW/cm^2^, the attenuation approaches saturation, enabling maximum modulation. This attenuation is attributed to THz absorption and reflection by photogenerated carriers [40,41].

**Figure 5:**
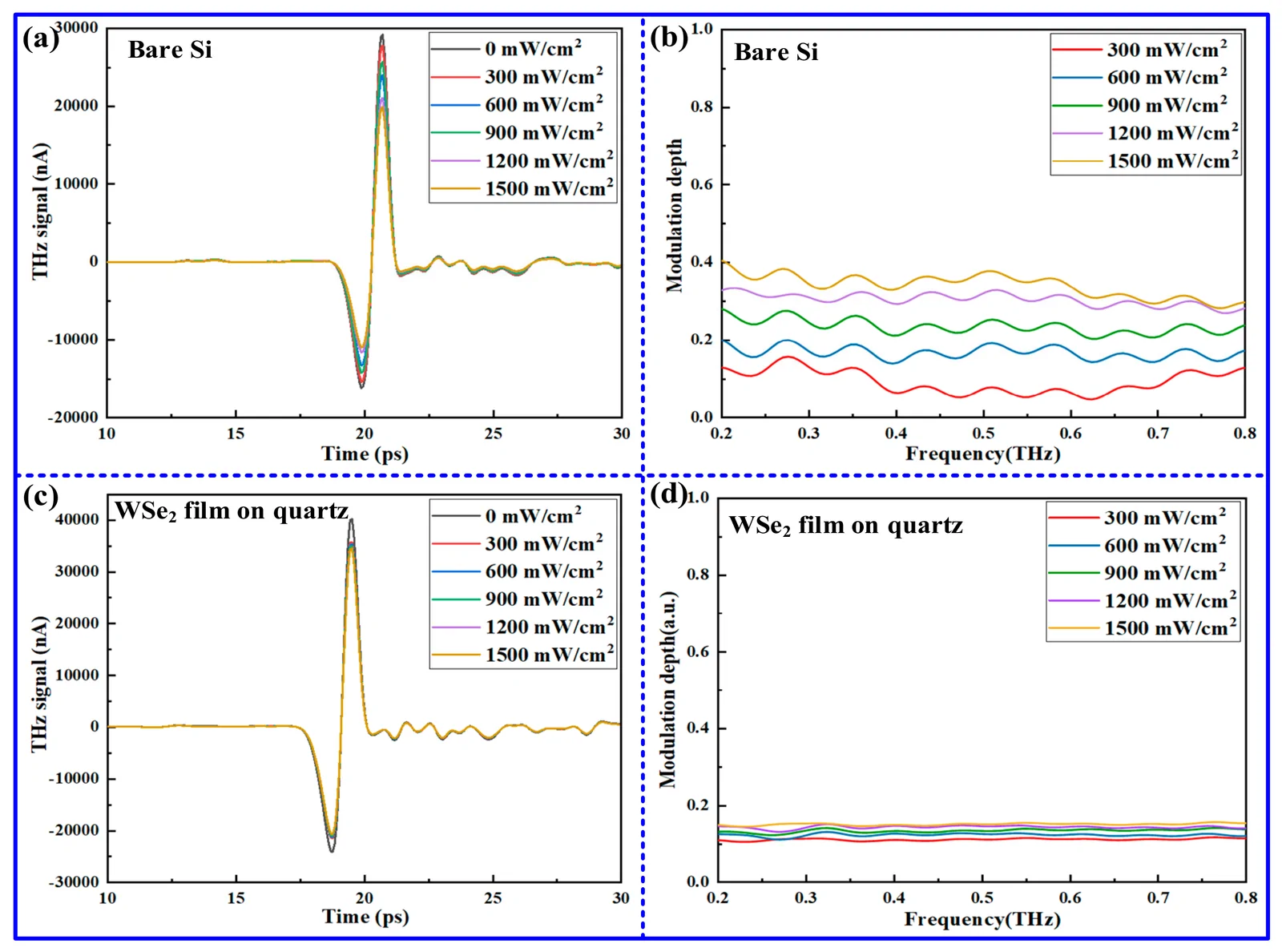
THz signals and modulation depths of bare Si and WSe_2_/quartz. (**a**) bare Si (time-domain), (**b**) bare Si (MD), (**c**) WSe_2_/quartz (time-domain), (**d**) WSe_2_/quartz (MD).

To quantify the modulation performance, the modulation depth (MD) is defined as [42]:

(2) $$MD=\frac{\int P_{\mathrm{pump-off}}\;(\omega )d\omega -\int P_{\mathrm{pump-on}}\;(\omega )d\omega }{\int P_{\mathrm{pump-off}}\;(\omega )d\omega }$$

where $P_{\mathrm{pump-off}}\;(\omega )$ and $P_{\mathrm{pump-on}}(\omega )$ denote the THz amplitudes without and with pumping. Figure 4e shows the MD across 0.1–1.1 THz at different pump powers. The MD increases progressively with the increase in pump power from 300 to 1200 mW/cm^2^ and saturates above 1200 mW/cm^2^. At 1500 mW/cm^2^, a maximum MD of 72% is achieved at 0.5 THz. For comparison, Figure 5 shows the time-domain THz transmissions and MDs for bare Si and a WSe_2_ film on quartz under identical pumping. The WSe_2_/quartz sample exhibits much stronger time-domain signals; yet, its maximum MD is only 15%, compared with 40% for bare Si and 72% for WSe2/Si heterojunction. Thus, the heterojunction modulator achieves an MD approximately two times that of bare Si and five times that of the WSe_2_/quartz, highlighting the critical role of the heterojunction in boosting MD. Additionally, it is worth noting that the MD values in Figure 4e and Figure 5 are compared using incident pump power densities, a common practice in the literature [41,43]. However, the absorbed pump power may differ among samples owing to their distinct absorption at 850 nm. In the WSe_2_/Si heterojunction, the 500 nm WSe_2_ layer absorbs only a small fraction of the incident 850 nm light, because the pump penetration depth in Si (≈13 μm) is much larger than the WSe_2_ thickness. Consequently, the pump absorption is dominated by the Si substrate and is essentially the same as that of bare Si. Therefore, the direct comparison between WSe_2_/Si and bare Si remains valid. For the WSe_2_/quartz, the WSe_2_ film is the sole absorber, and its absorption at 850 nm is substantially lower than that of Si, so its effective absorbed pump power is much smaller, partially accounting for its low MD. Nevertheless, even after considering for the absorbed power, the WSe_2_/Si heterojunction still exhibits a markedly higher MD than bare Si, because the heterojunction enhances carrier lifetime and conductivity change, as discussed below. Hence, our main conclusion, that the heterojunction significantly boosts modulation performance, remains robust.

Figure 4f shows the peak-to-peak time-domain signal as a function of pump power, displaying an approximately linear decrease with the increase in pump power from 0 to 1500 mW/cm^2^, indicating significant THz attenuation. Figure 4g plots the normalized transmission intensity versus pump power at 0.5 and 1.0 THz. Without pumping, transmission exceeds 0.88 at both frequencies, and at 1500 mW/cm^2^, it decreases to 0.28 (0.5 THz) and 0.17 (1.0 THz). The nearly identical trend confirms broadband operation. Figure 4h shows the MD dependence on pump power at same frequencies. With the increase in pump power, the MD increases rapidly and then saturates, reaching maximum values of 0.72 (0.5 THz) and 0.62 (1.0 THz) at 1500 mW/cm^2^. Compared with previously reported structures, moreover, our device exhibits a wide broadband and high MD, as shown in Table 3.

**Figure 6:**
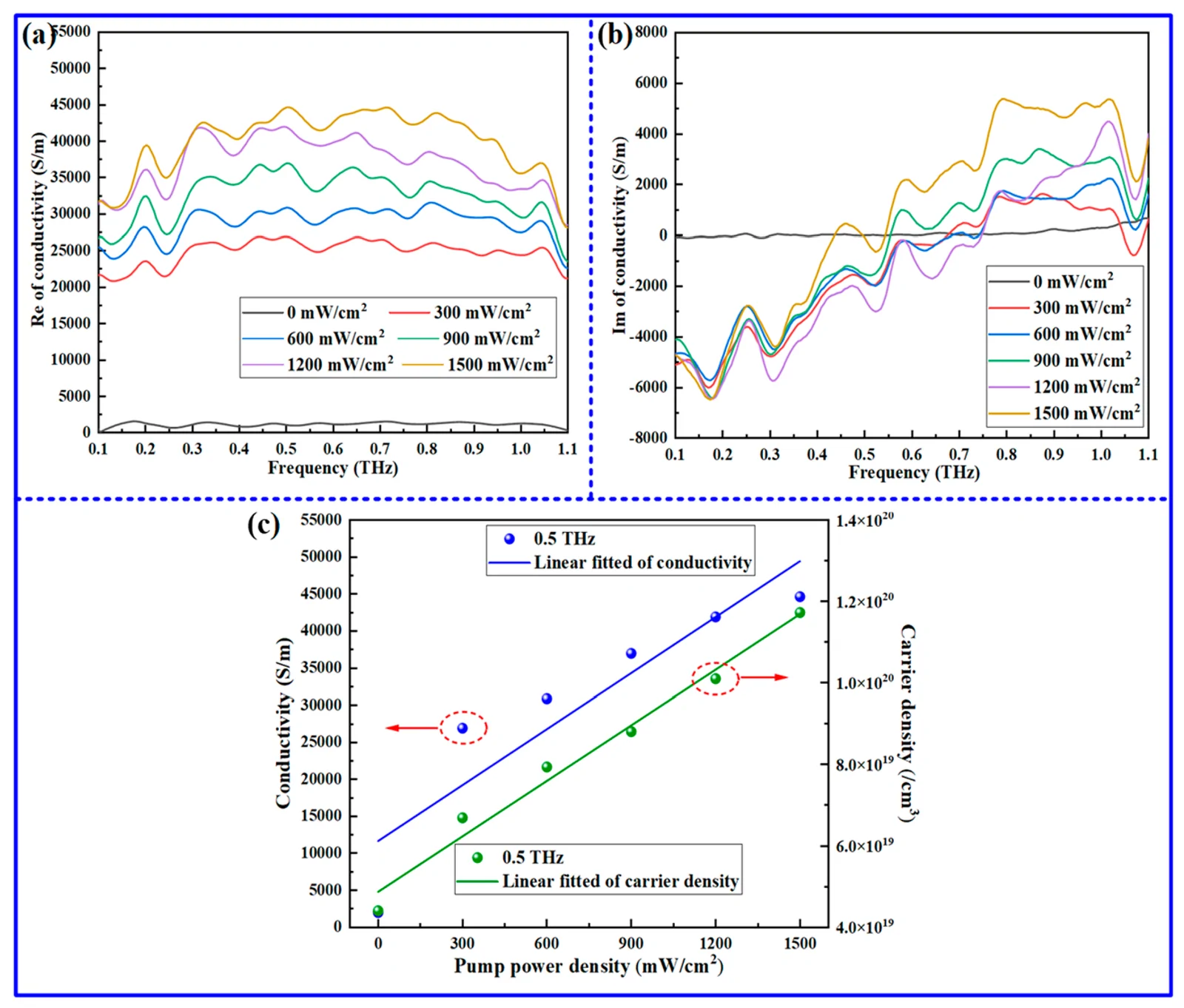
(**a**) Real part and (**b**) imaginary part of the complex conductivity of the WSe_2_/Si heterojunction, and (**c**) conductivity and carrier density as functions of pump power density.

To elucidate the mechanism, we extracted the photo-induced complex conductivity from the measured complex transmission $\tilde{T}(\omega )$ using Tinkham’s equation [45,46]:

(3) $$\tilde{\sigma }(\omega )\;=\;\frac{n_{\mathrm{sub}}+1}{Z_{0}d}\;(\frac{1}{\tilde{T}(\omega )}-1)$$

where $Z_{0}\;=\;377\;\Omega$ is the free-space impedance, *d* is the effective photoconductive layer thickness, and $n_{\mathrm{sub}\;}=\;3.42$ is the refractive index of Si. Hence, the MD is mainly determined by the conductivity, which varies with the carriers’ dynamics. Under 850 nm CW pumping, it is important to quantitatively assess the pumping absorption from the 500 nm WSe_2_ film or/and the Si substrate. As discussed above, since quartz is insulating and generates no photocarrier, the WSe_2_/quartz sample achieves only 15% MD due to only 500 nm thickness, while bare Si reaches 40%. The low MD can be attributed that the 500 nm WSe_2_ film is too thin to produce enough photocarriers by itself. Hence, the conductivity change in the heterojunction arises from photogenerated charge carriers in both silicon and WSe_2_. However, the WSe_2_ film is much thinner than Si and absorbs only a minor fraction of the pumping light. The Si absorbs most of the laser power, with the majority of carriers generated in the penetration depth (≈13 μm). Thus, *d* is interpreted as the characteristic spatial extent of the photogenerated carrier population, i.e., the pump penetration depth in Si [47], and the thin-film condition (*d* ≪ λ) holds across 0.1–1.1 THz [48]. Figure 6 presents the real and imaginary parts of the complex conductivity as functions of frequency at different pump powers. The real part (Figure 6a) increases significantly with pump power, reducing the transmission and enhancing MD. In contrast, the imaginary part (Figure 6b), associated with phase modulation [49], exhibits only weak pump-power dependence, consistent with the negligible time shift observed in the inset of Figure 4b. These results indicate that the modulator is primarily suited for amplitude modulation.

To further quantify the carrier contribution, the complex conductivity was fitted using the Drude model [50]:

(4) $$\tilde{\sigma }(\omega )=\frac{\varepsilon_{0}\omega_{p}^{2}}{\Gamma -i\omega }(1+\frac{C}{1-i\omega /\Gamma })$$

where *ε*_0_, *Γ*, *ω*_p_ and C are the vacuum permittivity, scattering rate, plasma frequency, and the backscattering coefficient, respectively. The plasma frequency relates to the charge carrier density $N_{c}$ via $\omega_{p}^{2}=N_{c}e^{2}/{(m}_{\mathrm{eff}}\varepsilon_{0})$, where e is the electron charge and $m_{\mathrm{eff}}$ is the effective mass. Figure 6c plots the conductivity and carrier density at 0.5 THz as functions of pump powers. Without pumping, the conductivity is near zero due to thermal equilibrium carriers. Upon excitation, both quantities increase linearly with pump power, reaching maximum values at 1500 mW/cm^2^, further confirming efficient modulation via pump intensity. Notably, the near-linear increase in extracted conductivity and carrier density does not contradict the apparent saturation of THz transmission and modulation depth at high pump powers (Figure 4e,f). This seeming discrepancy is reconciled by the nonlinear THz transmission–photoconductivity relationship inherent in the Tinkham’s equation [51]: the normalized transmission is given by $T(\omega )\;={\left|1+Z_{0}d\sigma (\omega )\right|}^{-2}$. In the low-excitation regime ($Z_{0}d\sigma (\omega )$ ≪ 1), *T* decreases approximately linearly with increasing σ. At higher pump powers, however, $Z_{0}d\sigma (\omega )$ becomes comparable to or greater than unity. Consequently, further increases in *σ* yield progressively smaller reductions in *T*, resulting in the observed saturation. Additionally, at high injection levels, Auger [52] and Shockley–Read–Hall [53] recombination become increasingly dominant, leading to a pump-dependent effective lifetime and a transition of the carrier density from linear to sublinear dependence on pump power [54]. Thus, the combined effect of the intrinsic Tinkham nonlinearity and high-injection recombination fully accounts for the saturation of the macroscopic THz response, while the linear fitting in Figure 6c remains physically valid across the moderate pump-power range (0–1500 mW/cm^2^), where linear recombination prevails.

**Figure 7:**
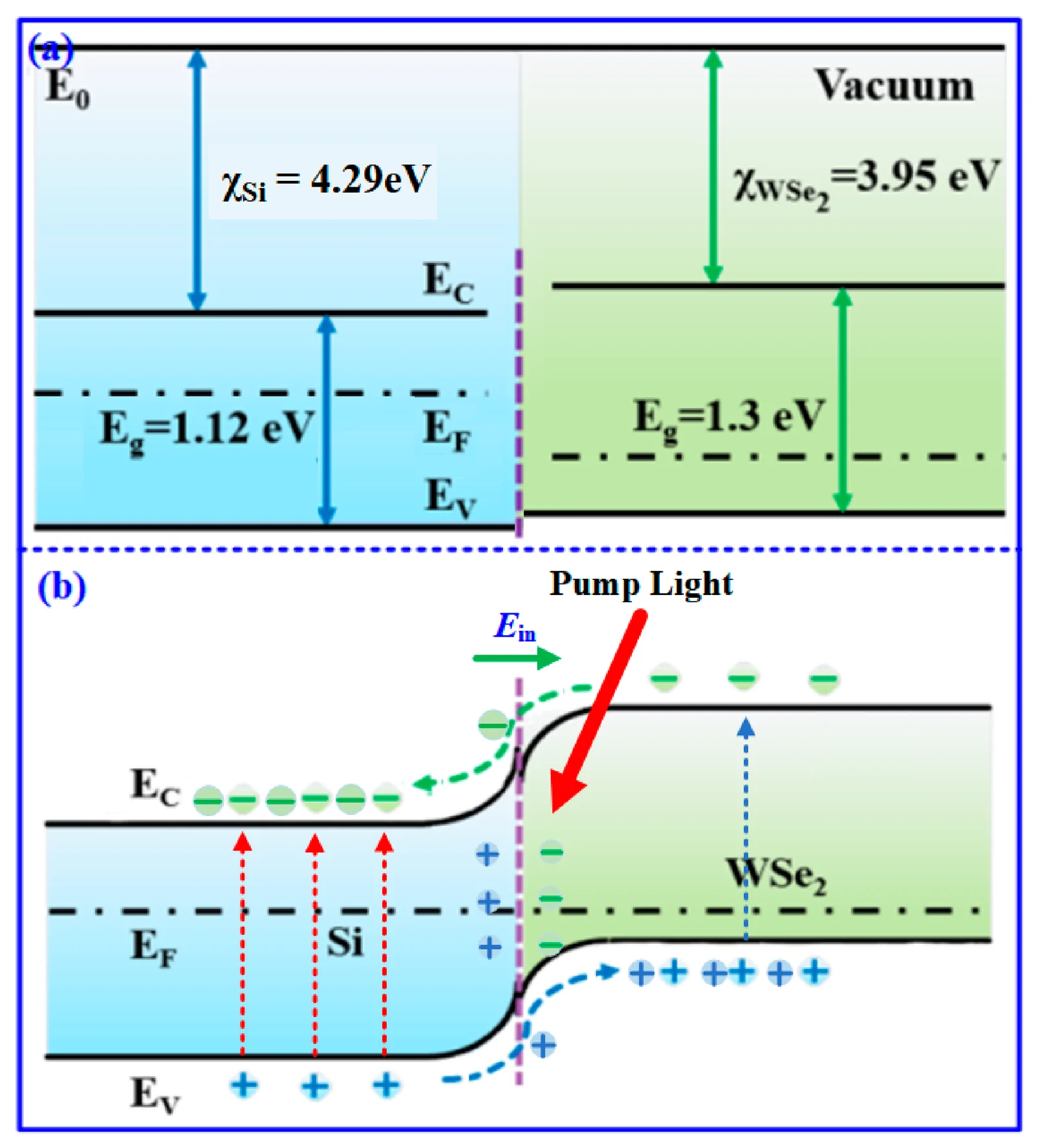
Band schemes of the WSe_2_/Si system: (**a**) energy bands of WSe_2_ and Si before contact and (**b**) band bending after contact and photogenerated carrier flow.

To further elucidate the enhanced conductivity and carrier density, the band alignment of the WSe_2_/Si heterojunction is shown in Figure 7a. Owing to the different bandgaps and Fermi levels in Si and WSe_2_, the conduction-band minimum (CBM) and valence-band maximum (VBM) are unaligned, creating an interfacial electric field. The electron affinity and bandgap differences yield a conduction-band offset of 0.05 eV and a valence-band offset of 0.13 eV, forming a type-II band alignment [55]. In high-resistivity Si (resistivity > 5000 Ω·cm and carrier concentration < 10^13^ cm^−3^), the Fermi level *E_F_* lies near midgap, while in WSe_2_, it lies close to the valence band, and is thus lower than that in Si. Upon contact, electrons diffuse from Si to WSe_2_ and holes in the opposite direction, establishing a built-in electric field (*E*_in_) oriented from Si to WSe_2_ at equilibrium, as illustrated in Figure 7b.

Under CW illumination, photocarriers are generated in both materials via interband transitions. However, because Si absorbs most of the pump power, it generates abundant photogenerated carriers, while WSe_2_ contributes only a few in WSe_2_. Moreover, the mobilities of the photogenerated electrons and holes in WSe_2_ are significantly lower than those in Si [56]. Therefore, the conductivity change in the heterojunction is dominated by the photocarriers in Si:

(5) $$\Delta \sigma \;=\;e(\mu_{n}\Delta n\;+{\;\mu }_{p}\Delta p)$$

where *μ*_n_ and *μ*_p_ are the electron and hole mobilities in high-resistivity Si (1450 and 500 cm^2^/V·s, respectively), and Δ*n* and Δ*p* are the non-equilibrium carrier concentrations. Additionally, the recombination probability and lifetime *τ* of non-equilibrium electrons depend mainly on Δ*p*, with *τ* ∝1/Δ*p* [57]. Under the built-in electric field, photogenerated electrons transfer from WSe to Si while holes move in the opposite direction, enabling efficient separation of electrons and holes, as shown in Figure 7b. The separation reduces Δ*p* in Si and suppresses electron-hole recombination, thereby prolonging the effective electron lifetime *τ*, increasing Δ*n* in Si, and enhancing the MD. Since *μ*_n_ in high-resistivity Si is approximately three times *μ*_p_, the Δ*σ* is dominated by the enhanced electron population, leading to decreased THz transmission and increased MD [36]. Thus, the WSe_2_ layer acts not as a primary photoconductive channel but as an interfacial engineering layer that modulates carrier dynamics in Si and serves as a minority-carrier trap or surface-state mediator, similar to passivation layers in conventional semiconductor heterojunctions [28,29]. Thus, the enhanced modulation originates from interface-induced modifications to carrier dynamics in Si, not from direct photoconductive contributions of the WSe_2_ layer itself. In addition, the uneven surface morphology (Figure 2e,f) may enhance THz radiation trapping via an anti-reflection effect [58], further contributing to the large MD.

Additionally, the proposed band-alignment mechanism for enhancing the MD is further substantiated by recent experimental observations [59]. For example, time-resolved THz emission spectroscopy on monolayer WSe_2_/Si heterojunctions directly reveals an interface-induced drift-current amplification of up to one order of magnitude, demonstrating that the built-in electric field accelerates carriers migration and enhances photocurrent [36]. Concurrently, optical pump-terahertz probe (OPTP) measurements on pristine WSe_2_ show that photocarriers are generated on a sub-picosecond timescale and exhibit relaxation lifetimes of several hundred picoseconds, confirming that the intrinsic carrier dynamics in WSe_2_ enable ultrafast response while remaining long-lived enough to contribute meaningfully to the photoconductivity changes [60]. Collectively, these observations corroborate the proposed mechanism: the interfacial built-in electric field spatially separates electrons and holes, suppresses carrier recombination, prolongs the effective carrier lifetime, and thereby enhances net photoconductivity and THz MD.

Beyond modulation depth, modulation speed is a critical parameter for practical applications. Although our CW-pumping THz-TDS system is configured for steady-state transmission measurements and does not directly provide the frequency-dependent modulation response, the intrinsic carrier dynamics offer a reliable estimate for the response time. Using measured carrier lifetimes (τ ≈ 100–500 ps) reported in [60] and the interface-enhanced carrier separation described in [36], we estimate the intrinsic 3 dB modulation bandwidth of our WSe_2_/Si heterojunction modulator to be on the order of 1–2 GHz (BW ≈ 1/(2πτ)). A comprehensive experimental characterization of the modulation speed as a function of the chopping frequency will be pursued in future work, since the practical speed is also influenced by the chopper settings and external circuit parasitics.

## 4. Conclusions

In summary, we have successfully fabricated a large-area WSe_2_/Si heterojunction-based THz modulator with large MD using magnetron sputtering and vacuum selenization. The device exhibits a broadband response from 0.1 to 1.1 THz under 850 nm CW laser pumping. At a pump power density of 1500 mW/cm^2^, a maximum modulation depth of 72% is achieved at 0.5 THz, significantly surpassing those of bare Si and single WSe_2_ films. The enhanced performance is attributed to the heterojunction-induced built-in electric field, which effectively separates photogenerated carriers and suppresses recombination, thereby amplifying the conductivity change. Notably, compared with most reported THz modulators based on CVD-grown 2D materials, our heterojunction offers competitive modulation performance while being amenable to large-area fabrication. Therefore, this work provides a practical and scalable pathway for realizing effective THz modulation using large-area 2D film materials.

## Figures and Tables

**Table 1:** Parameters of magnetron sputtering.

Parameter	Value
Target purity	99.99%
Working pressure	2 Pa
Background vacuum	6.0 × 10^−4^ Pa
Sputtering power	25 W
Ar flow	5.4 sccm
Target distance	10 cm
Deposition time	5 min

**Table 2:** Parameters of selenization reaction.

Parameter	Value
Purity of Se powder	99.99%
Se powder content	100 mg
Ar flow	30 sccm
Reaction time	3 h
Heating rate	5 °C/min
Reaction temperature	850 °C

**Table 3:** Comparison of modulation performance of previously reported THz modulators.

Structures	Modulation Bandwidth (THz)	Modulation Depth (%)	Pumping Wavelength (nm)	Fabrication Method	Ref.
PtSe_2_/Si	0.1–1.0	32.7	800	Sputtering + selenization	[31]
GeSe_2_/Si	0.2–1	60	532	Sputtering + selenization	[32]
TaS_2_/Si	0.2–1.0	46.8	800	Sputtering + sulfurization	[40]
MoTe_2_/WTe_2_	/	7.5	800	CVD	[44]
WSe_2_/Si	0.1–1.1	72	850	Sputtering + selenization	This work

## Data Availability

The original contributions presented in this study are included in the article. Further inquiries can be directed to the corresponding authors.
